# TDM-Guided Dalbavancin Treatment for Complex *Staphylococcus aureus* Osteoarticular Infections in Children

**DOI:** 10.3390/antibiotics15020162

**Published:** 2026-02-03

**Authors:** Silvia Garazzino, Giulia Mazzetti, Matteo Sandei, Raffaele Vitale, Camilla Martino, Alice Palermiti, Amedeo De Nicolò, Elisa Funiciello, Alessandro Aprato, Alessia Gerace, Alessandro Bondi, Antonio Curtoni, Antonio D’Avolio, Marco Denina

**Affiliations:** 1 Infectious Diseases Unit, Department of Public Health and Pediatrics, University of Turin, Regina Margherita Children’s Hospital, 10126 Turin, Italy; 2 Department of Public Health and Pediatrics, Postgraduate School of Pediatrics, University of Turin, 10124 Turin, Italy; 3 Department of Public Health and Pediatrics, University of Turin, 10126 Turin, Italy; 4 Laboratory of Clinical Pharmacology and Pharmacogenetics, Department of Medical Sciences, University of Turin, 10149 Turin, Italy; 5 Pediatric Onco-Hematology Unit, Regina Margherita Children’s Hospital, 10126 Turin, Italy; 6 Paediatric Orthopaedics and Traumatology Unit, Department of Surgical Sciences, University of Turin, Regina Margherita Children’s Hospital, 10126 Turin, Italy; 7 Microbiology and Virology Unit, A.O.U. Città Della Salute e Della Scienza di Torino, 10126 Turin, Italy; 8 Microbiology and Virology Unit, Department of Public Health and Pediatrics, University of Turin, A.O.U. Città Della Salute e Della Scienza di Torino, 10126 Turin, Italy; 9 Infectious Diseases Unit, Regina Margherita Children’s Hospital, 10126 Turin, Italy

**Keywords:** dalbavancin, pharmacokinetics, therapeutic drug monitoring, children

## Abstract

**Background/Objectives**: Dalbavancin is approved for pediatric acute bacterial skin and skin structure infections (ABSSSIs), yet real-world practice frequently necessitates off-label use for deep-seated infections requiring prolonged suppression. While adult data support therapeutic drug monitoring (TDM)-guided maintenance, the pediatric evidence for repeated-dose pharmacokinetics (PK) is limited. We evaluated the efficacy, safety, multi-dose PK, and pharmacoeconomic impact of dalbavancin in a complex pediatric cohort. **Methods**: A retrospective study (2023–2025) of enrolled patients < 18 years treated with dalbavancin. A subgroup receiving ≥3 doses underwent PK analysis to assess concentration decay against conservative efficacy targets (4 and 8 mg/L). A pharmacoeconomic analysis compared resource utilization against the standard of care. **Results**: Sixteen patients (median age 12) were included, primarily treated for *Staphylococcus aureus (S. aureus*) osteoarticular infections (75%), and frequently device-associated (66.7%). Clinical success was 93.8% (15/16) with no adverse events. A PK analysis (*n* = 9; 78 samples) ruled out dangerous accumulation but revealed a significant concentration drop at week 4 (mean 6.06 mg/L; *p* = 0.005). Logistic regression identified the time since the previous dose as the sole predictor of sub-therapeutic levels, with >50% of the patients dropping below 8 mg/L by the fourth week. An analysis showed median net savings of EUR 3215.84 per patient (*p* = 0.004). **Conclusions**: Dalbavancin is effective and cost-saving for complex pediatric infections. However, due to distinct pediatric PK, dosing regimens extrapolated from adults may result in sub-therapeutic concentrations by week 4. We recommend TDM around week 3 to tailor dosing or limiting maintenance intervals to a maximum of 4 weeks.

## 1. Introduction

Infections caused by Gram-positive bacteria, particularly *Staphylococcus aureus*, represent a leading cause of pediatric morbidity. The clinical spectrum ranges from acute bacterial skin and skin structure infections (ABSSSIs) to invasive and deep-seated conditions, such as osteoarticular infections (OAIs) and prosthetic joint infections (PJIs), which account for a significant and increasing volume of hospitalizations [1,2,3,4]. Traditionally, the effective management of these infections relies on prolonged intravenous (IV) antibiotic courses, often necessitating extended hospitalization and the placement of central venous catheters (CVCs) [1,2,3,4]. Such requirements impose a heavy economic burden and increase the risk of adverse events, particularly CVC-related complications, drug toxicity, and nosocomial infections [5,6]. Moreover, even oral step-down options are often limited in pediatrics by poor palatability, lack of age-appropriate formulations, and adherence challenges over prolonged courses [7].

In this landscape, dalbavancin has emerged as a valuable therapeutic tool [8]. As a long-acting lipoglycopeptide with potent bactericidal activity against Gram-positive pathogens, high efficacy against biofilm, and excellent bone penetration, it offers an alternative to both daily IV regimens and prolonged oral therapy [9,10,11,12]. Dalbavancin was first approved by the Food and Drug Administration (FDA) and the European Medicines Agency (EMA) in 2014 and 2015, respectively, for the management of ABSSSIs in adults, using a total dose of 1500 mg administered either as a single infusion or as a split two-dose regimen. Subsequently, between 2021 and 2022, both the FDA and the EMA extended the indication to ABSSSIs in pediatric patients aged at least 3 months, with a single-dose regimen of 22.5 mg/kg (<6 years) or 18 mg/kg (≥6 years), establishing dalbavancin as the first long-acting antibiotic in pediatrics [13,14].

However, real-world clinical practice often extends beyond the boundaries of the approved label [12,15,16,17]. Clinicians frequently face complex scenarios, such as OAIs or complicated ABSSSIs, where the dalbavancin standard single-dose therapeutic regimen is insufficient [17,18,19,20]. The literature for adult data largely support the off-label use of dalbavancin for osteomyelitis and PJIs, providing defined dosing strategies for prolonged multi-dose treatment regimens and identifying therapeutic drug monitoring (TDM) as a fundamental tool to ensure optimal drug exposure [10,19,21,22,23,24]. Conversely, the pediatric evidence in this setting is very limited. Substantial knowledge gaps remain regarding the pharmacokinetics of repeated doses in children and the correct timing of multi-dose regimens; no data are available regarding the use of TDM in the pediatric population [16]. Dalbavancin displays high protein binding (~93%) and significant renal elimination (33–50%) [25,26]. Pediatric physiology, characterized by renal maturation and a larger relative volume of distribution, results in intrinsically higher weight-normalized clearance compared to adults [27]. In critically ill children, this rapid elimination mechanism may be further exacerbated by the phenomenon of augmented renal clearance (ARC) [28]. Consequently, given these distinct pharmacokinetic profiles, it is unclear whether dosing intervals extrapolated from adult protocols are adequate to maintain therapeutic concentrations over time [29,30,31]. Specifically, questions remain regarding the ability to sustain levels above 4 mg/L and 8 mg/L, considered respectively the minimum and the conservative thresholds to ensure efficacy and to prevent resistance selection in deep-seated staphylococcal infections [18,32]. Furthermore, the pharmacoeconomic impact of switching from standard inpatient care to dalbavancin-based management in a pediatric setting has yet to be fully quantified [33].

The aim of this study is to evaluate the real-life clinical efficacy and safety of dalbavancin in a complex pediatric cohort. To this end, we performed a detailed pharmacokinetic (PK) analysis to define the optimal dosing strategy for prolonged regimens and to assess the role of TDM in the pediatric population. Additionally, through a pharmacoeconomic analysis, we evaluated the cost-effectiveness of this approach.

## 2. Results

A total of 16 patients were enrolled in this study. The demographic and clinical findings are detailed in Table S1. The cohort showed an equal gender distribution and a median age of 12 years (interquartile range (IQR) 8–15). Osteoarticular infections were the main indication (75%), with a high prevalence of device-associated cases (66.7% of osteoarticular infections).

*Staphylococcus aureus* was the predominant pathogen (81%). Notably, the dalbavancin minimum inhibitory concentration (MIC) values were not available as specific susceptibility testing is not routinely performed in our local laboratory.

As shown in Table S1, dalbavancin was primarily used as a consolidation therapy (93.8% of cases) following a median of 22.4 days (IQR 13.0–31.3) of previous antibiotic treatment. Most patients (62.5%) underwent a prolonged dosing regimen (up to 8 doses), while the others received a short course (1–2 doses).

Overall, therapy was well-tolerated with no adverse events. Clinical efficacy was 93.8% (15/16). The single case marked as ‘Failure’ in Table S1 refers to a patient who experienced a recurrence attributed to the incomplete sterilization of the osteosynthesis devices. However, following this event, the patient was successfully retreated with a second course of dalbavancin, achieving a positive therapeutic outcome.

### 2.1. Pharmacokinetics Analysis and TDM

A pharmacokinetic analysis was conducted on the TDM subgroup (*n* = 9). All patients included in this cohort were treated for osteoarticular infections with *S. aureus* as the main etiological pathogen (*n* = 8/9; 88.9%). The demographic data, dosing regimens, and TDM details are summarized in Table 1, while specific clinical and microbiological characteristics for each patient can be cross-referenced in Table S1 using the corresponding patient ID.

Note regarding renal function: The eGFR was calculated using the pediatric bedside Schwartz formula. Patient 1 showed normal renal functions (89.29 mL/min/1.73 m^2^), whereas values > 110–130 mL/min/1.73 m^2^ in the other patients are consistent with augmented renal clearance (ARC), a hyper-filtration response common in pediatric systemic infections.

**Figure 1:**
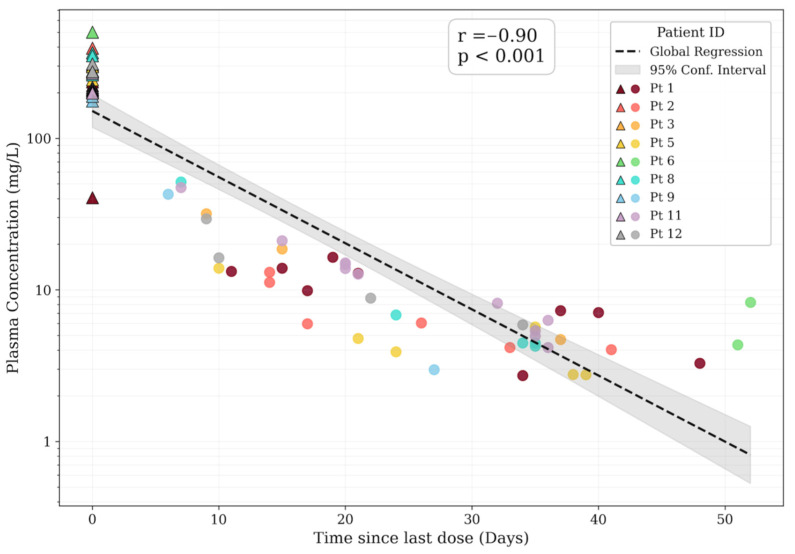
Semilogarithmic plot of dalbavancin plasma concentration–time profile. The observed serum concentrations are stratified by patient ID (color-coded). Triangles indicate peak levels (time 0 post-infusion), while circles represent subsequent trough levels. The dashed black line represents the global log-linear regression fit. The grey shaded area indicates the 95% confidence interval of the regression line. The linear regression indicates a highly significant negative correlation (*p* < 0.001, *r* = −0.90), demonstrating the expected pharmacokinetic decay during the dosing interval.

A total of 78 serum samples were collected. An analysis of the full TDM dataset demonstrated a highly consistent concentration–time profile. A global regression analysis revealed a strong log-linear decay pattern relative to the time elapsed since infusion (*r* = −0.90, *p* < 0.001; Figure 1). This confirmation of predictable first-order elimination kinetics validates the internal consistency of the dataset for the subsequent stability and target attainment analyses.

**Figure 2:**
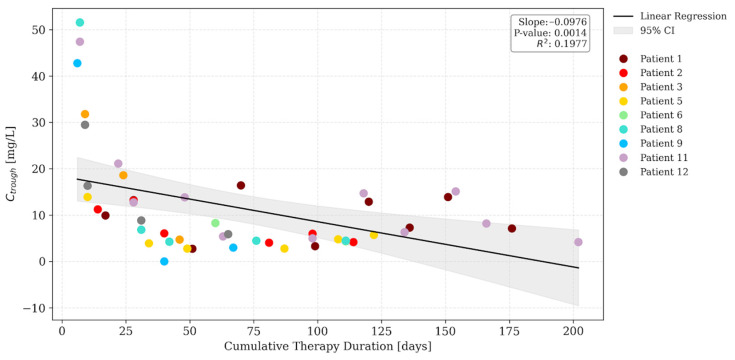
Linear regression analysis of trough concentrations versus cumulative duration of therapy (all samples). Colored dots represent individual patient samples, the solid black line indicates the linear regression fit, and the grey shaded area represents the 95% confidence interval (95% CI).

**Figure 3:**
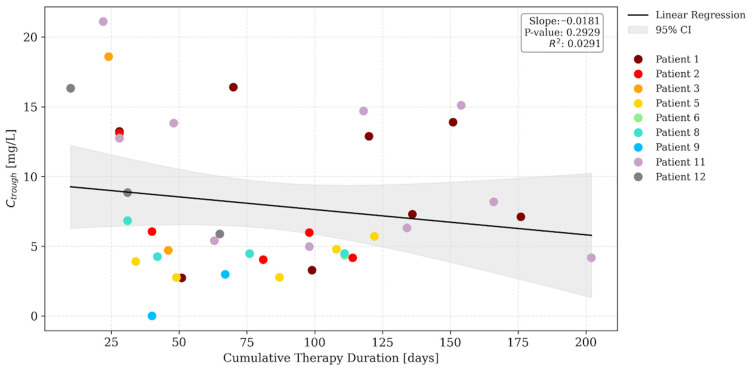
Linear regression analysis of trough concentrations excluding the first dose samples (dose ≥ 2 samples). Colored dots represent individual patient samples, the solid black line indicates the linear regression fit, and the grey shaded area represents the 95% confidence interval (95% CI). The linear regression line is displayed to illustrate the trend of trough concentrations over time. The non-significant regression indicates a stable trend (stationarity), confirming that no significant drug accumulation occurred despite the increasing duration of therapy.

The relationship between dalbavancin trough concentrations and cumulative therapy duration was initially evaluated via linear regression across the entire dataset. While a significant negative slope was observed (*slope* = −0.098; *p* = 0.001; Figure 2), this was identified as a sampling timing bias. Dose 1 samples were indeed clustered in the early distribution phase, whereas subsequent doses were sampled at longer intervals relative to the previous administration. To correct for this, a secondary analysis was conducted excluding Dose 1 samples. It revealed a stable pharmacokinetic profile with a non-significant slope (*slope* = −0.018; *p* > 0.05; Figure 3), confirming pharmacokinetic stationarity. This demonstrates that drug exposure remains consistent over time, without evidence of progressive accumulation or changes in clearance during prolonged therapy.

**Figure 4:**
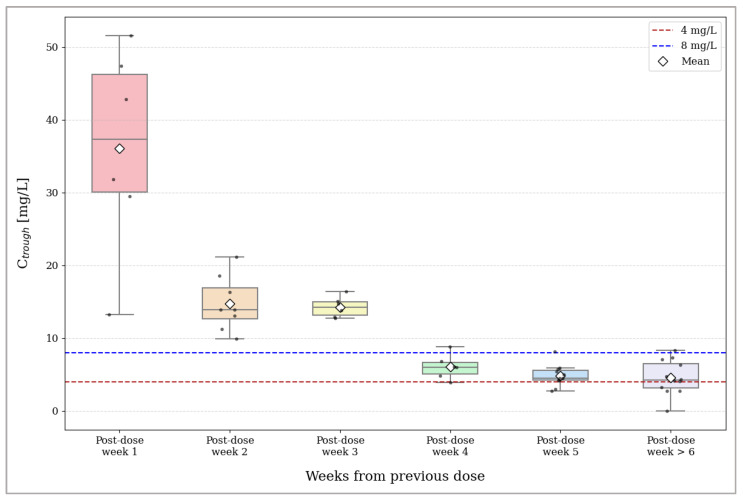
Distribution of intra-cycle dalbavancin trough plasma concentrations stratified by post-dose time. Boxplots represent the distribution of serum levels (mg/L) observed from week 1 to ≥week 6 post-dose. Dashed horizontal lines mark clinical reference thresholds: the optimal coverage threshold at 8 mg/L (blue) and the minimum threshold at 4 mg/L (red).

Based on this stationarity, samples were grouped by weeks elapsed since the previous dose to analyze the intra-cycle decay (Table 2 and Figure 4). Post-dose week 2 was selected as the reference baseline to mark the onset of the terminal elimination phase (Table 3). The analysis identified two distinct kinetic phases:

- Therapeutic stability (post-dose weeks 2–3): Concentrations remained high and stable, ranging from 14.76 mg/L to 14.28 mg/L. Post-dose week 3 showed the lowest inter-individual variability (coefficient of variation (CV) = 9.80%), and paired *t*-tests confirmed no statistically significant difference compared to week 2 (*p* = 0.843);
- Limit of optimal coverage (post-dose week 4 onwards): A critical breakpoint was identified starting from the fourth week, marking the limit for optimal coverage (defined as concentration < 8 mg/L). A significant reduction in concentration was detected at post-dose week 4 (mean 6.06 mg/L; *p* = 0.005). This significant decline persisted in later phases (post-dose week 5 and ≥6: *p* = 0.001), accompanied by a progressive increase in variability (CV up to 50.98%).

Two binomial logistic regression models were fitted to identify the predictors of sub-therapeutic concentrations, defined as levels falling below the thresholds of 4 mg/L (Table 4) and 8 mg/L (Table 5). Logistic regression identified the time elapsed since the previous infusion (“C_trough_ timing”) as the only statistically significant predictor of falling below the therapeutic thresholds (*p* = 0.015, OR: 2.96 for <4 mg/L; *p* < 0.001, OR 3.05 for <8 mg/L). Other variables, such as eGFR, weight-adjusted dose, and total days of therapy, were not statistically significant (*p* > 0.05).

**Figure 5:**
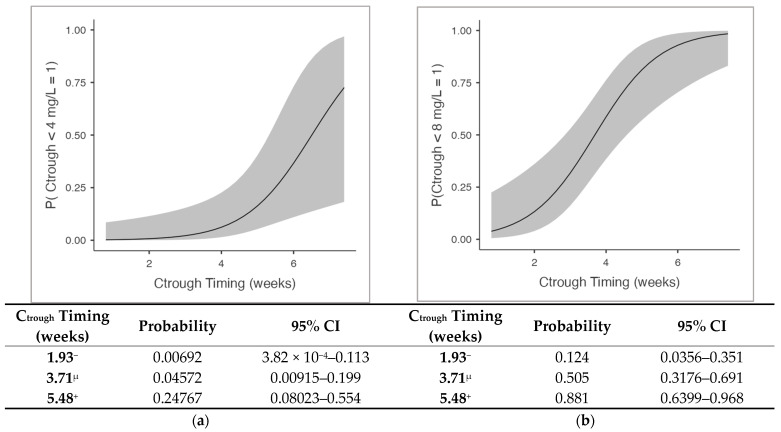
Logistic regression analysis predicting the probability of sub-therapeutic dalbavancin exposure over time: (**a**) Predicted probability of trough concentrations (C_trough_) falling below the 4 mg/L threshold as a function of time elapsed since the previous dose; (**b**) Predicted probability of C_trough_ falling below the 8 mg/L threshold. In both panels, the solid black line represents the estimated probability, while the grey shaded area indicates the 95% confidence interval (95% CI). The tables below the plots display specific probability estimates at representative time points. Symbols in the table: ‘μ’, mean value of the covariate; ‘^−^’, mean minus 1 standard deviation (SD); ‘^+^’, mean plus 1 standard deviation (SD).

An analysis of the EMMs revealed a risk escalation over time (Figure 5): at 2 weeks post-infusion, exposure is robust with a negligible risk of falling below 4 mg/L (0.07%) and a low risk for the 8 mg/L target (12.4%). A critical gap emerges by 4 weeks post-infusion, where half of the patients (50.5%) fail to maintain the 8 mg/L target despite a still-low risk for the 4 mg/L threshold (4.6%). By 5.5 weeks, the failure rates escalate significantly, with 24.8% and 88.1% of the patients dropping below 4 mg/L and 8 mg/L, respectively.

### 2.2. Optimization Resource Analysis

The economic analysis (*n* = 16) confirmed statistically significant benefits across all parameters, as detailed in Table 6. All data showed a normal distribution (Shapiro–Wilk test; all *p* > 0.05). Dalbavancin treatment significantly reduced hospital resource utilization, with a median of 8.5 days of hospitalization saved (*p* < 0.001) and a median reduction in CVC usage of 6.0 days (*p* = 0.009). From an economic perspective, these clinical efficiencies translated into substantial savings. The median gross hospitalization cost avoided was EUR 5852.25. Crucially, even after accounting for outpatient drug and administration costs, the final net saving remained positive and significant, with a median benefit of EUR 3215.84 per patient (*p* = 0.004) confirming the economic viability of the dalbavancin regimen despite the drug acquisition costs.

## 3. Discussion

This study provides a comprehensive real-life evaluation of dalbavancin in the pediatric population, integrating clinical efficacy, safety, TDM-guided pharmacokinetic analysis, and a detailed assessment of healthcare resource optimization.

### 3.1. Clinical Efficacy and Safety in the Pediatric Context

Although dalbavancin is approved in pediatrics primarily for ABSSSIs, our experience reflects an unsatisfied clinical need in the management of osteoarticular and complex infections [13,14,16]. The clinical success rate of 93.8% observed in our cohort is consistent with the data reported in recent systematic reviews on pediatric off-label use and aligns with the efficacy rates observed in adults with osteomyelitis [16,17].

The high complexity of our cohort, characterized by significant comorbidities and device-associated infections (66.7%), explains the prolonged hospitalization prior to dalbavancin (median 22 days). In this context, dalbavancin was utilized not as a first-line empirical choice, but as a strategic consolidation therapy (93.8% of cases). This strategy effectively facilitated discharge for clinically stable patients who would have otherwise required prolonged hospitalization or challenging treatments, aligning with recent real-world pediatric experiences [16,33]. From a microbiological perspective, while dalbavancin’s potent anti-biofilm activity likely contributed to success, the single failure involving a retained device confirms that pharmacological potency cannot substitute for adequate surgical source control [4,17].

A particularly relevant finding was the complete absence of adverse events (AEs), even in patients undergoing prolonged multi-dose regimens. This aligns with the favorable safety profile described in the literature, where pediatric trials show low AE rates (7.2–10.3%) [16]. This stands in contrast to the historical burden of standard long-term intravenous therapy for osteoarticular infections, which can affect up to 73% of children due to drug toxicity, nosocomial infections, or catheter-related complications [5,34].

### 3.2. Pharmacokinetic Analysis: Stationarity and the Week-4 Breakpoint

The most novel contribution of our work lies in the detailed pharmacokinetic analysis of repeated doses in children. While single-dose kinetics have been described, real-life data on prolonged pediatric regimens were virtually absent [29,30,31]. Contrary to the theoretical concerns regarding potential drug accumulation due to the long terminal half-life, our regression analysis demonstrated a clear stationarity of trough concentrations. This finding indicates that dalbavancin does not accumulate dangerously in plasma even after multiple administrations, validating the safety of long-term suppressive regimens in children, a concept recently supported by population modelling in adults but lacking clinical confirmation in pediatrics until now [20,21].

Regarding the intra-cycle behavior, our data revealed a profile consistent with the multiphasic kinetics described for dalbavancin [30]. This was characterized by an initial rapid decline (distribution phase), which explains the high dispersion of concentration values observed during the first week, followed by a slower terminal elimination phase. However, the analysis of the decay during this elimination phase revealed a crucial finding for clinical practice. We observed that concentrations remained stable above 14 mg/L from week 2 through week 3 post-infusion but then dropped significantly to a mean of 6.06 mg/L at week 4 (*p* = 0.005). This observed rapid decay contrasts with the adult pharmacokinetic profile, where concentrations typically remain high (>20 mg/L) for 4–5 weeks, supporting dosing intervals that could be extended up to 6–8 weeks [23,35,36]. Conversely, our findings align with pediatric single-dose PK studies demonstrating that distinct physiological characteristics, primarily enhanced renal clearance, drive faster drug elimination [30]. Although specific mass–balance studies in pediatrics are limited, the adult data confirm that renal excretion of the unchanged drug is a major elimination pathway (approx. 33–50%) [25]. Since glomerular filtration and tubular function mature to adult levels by the end of the first year of life, it is physiologically reasonable to assume that renal clearance remains the primary driver of elimination in our cohort, likely accelerated by the ARC phenomenon [27,28]. As described, this results in a reduction in total drug exposure ranging from approximately 30% in adolescents to ~40% in premature neonates compared to adults, shortening the effective therapeutic window [29,30,31]. Consequently, extended dosing intervals risk failing to ensure adequate efficacy, carrying a non-negligible probability of falling below the recommended thresholds for adult staphylococcal infections [18,32]. Specifically, our model estimates that, while the risk of falling below 4 mg/L is just 4.6% at 4 weeks, it rises to ~25% by 5.5 weeks; crucially, for the conservative 8 mg/L, the risk is already substantial (~50%) by week 4 post-infusion, reaching ~88% by week 5.5. This underscores the risk of treatment failure, particularly in deep-seated infections where bone penetration is critical.

### 3.3. The Role of TDM and the Predictors of Exposure

Our logistic regression identified the time elapsed since the previous dose as the only significant predictor of falling below therapeutic thresholds, whereas age, duration of therapy, weight-adjusted dose, and the eGFR did not show a significant impact. This confirms that, while weight-based dosing is appropriate for normalization, inter-individual variability becomes predominant as the dosing interval extends [18,19,29]. Notably, this is mirrored in our data by the coefficient of variation (CV) of plasma concentrations, which remained contained during the first weeks but increased markedly from week 4 post-infusion. This widening dispersion confirms that individual clearance rates, rather than initial dosing, predicted the exposure at the end of the dosing interval.

Consistent with the proactive TDM approach strongly recommended for multi-dose regimens in adults, our data support the implementation of TDM in children receiving long-term therapy [18,19,20,24]. Based on our EMM analysis, we suggest monitoring plasma levels around the third week post-infusion. If concentrations approach the 8 mg/L safety threshold, often cited as the conservative target for staphylococcal osteoarticular infections, the subsequent dose should be anticipated, rather than adhering rigidly to a fixed schedule [18,32].

### 3.4. Resource Optimization and Quality of Life

Beyond clinical efficacy, our study highlights how dalbavancin translates clinical efficiency into tangible economic sustainability, even with the multi-dose regimen. Our pharmacoeconomic analysis demonstrates that the reduction in healthcare resource utilization (median saving of 8.5 hospital days and 6 CVC days per patient) offsets the drug’s acquisition cost, resulting in a significant net saving of ~EUR 3200 per patient, confirming the potential to reduce the high economic burden associated with prolonged hospitalization described in the literature [5,37]. It is important to note that this saving was achieved in a “consolidation” setting, where dalbavancin was administered after a prolonged initial stabilization period. Theoretically, an earlier positioning of dalbavancin in the treatment algorithm (i.e., as first-line or early-switch therapy) could yield even greater economic benefits by considerably shortening the initial hospitalization phase and minimizing device-related risks.

Furthermore, minimizing the length of stay and the CVC duration generates other crucial benefits not captured by a direct cost analysis. Reducing hospital exposure directly lowers the risk of healthcare-associated infections, a complication that excessively affects complex pediatric patients [5]. Finally, from a social perspective, transforming inpatient care into outpatient management alleviates the burden on families, thereby reducing indirect costs such as parental work loss and school absenteeism, which are critical factors in pediatric care [15].

### 3.5. Limitations and Future Directions

Our study is not free of limitations. Firstly, it has a retrospective monocentric design and a small sample size for the PK subgroup (*n* = 9). Nevertheless, to the best of our knowledge, to date this study represents the first real-world description of repeated-dose kinetics in a pediatric cohort.

Secondly, the lack of specific MIC data was mitigated by adopting the conservative threshold of 8 mg/L (instead of the standard 4 mg/L). This conservative threshold minimizes the risk of overestimating therapeutic coverage, ensuring robustness even against strains with reduced susceptibility. Nevertheless, we acknowledge that applying these thresholds, as derived from adult pharmacokinetic/pharmacodynamic (PK/PD) studies, represents a necessary compromise in the absence of pediatric data. Consequently, establishing validated PK/PD ‘concentration–outcome’ correlations specifically for pediatric osteomyelitis constitutes a distinct and urgent priority for future research, essential to move beyond the reliance on adult surrogates.

Finally, we acknowledge the variability in sampling times. However, the internal consistency of the kinetic data and the statistical significance of the pharmacoeconomic analysis strengthen our conclusions.

Future research efforts should prioritize prospective, multicenter studies with larger cohorts to validate the PK stationarity observed here and to refine pediatric-specific dosing intervals.

## 4. Materials and Methods

### 4.1. Study Design and Population

This single-center, retrospective, observational study was conducted at the Regina Margherita Children’s Hospital in Turin (Italy). We consecutively enrolled all pediatric patients (aged < 18 years) who received at least one dose of dalbavancin for either on-label indications (ABSSSIs) or off-label use based on clinical judgment from October 2023 to October 2025. A specific subgroup was selected for the pharmacokinetic and TDM analysis (TDM subgroup). Inclusion in this subgroup was restricted to patients who received at least 3 consecutive doses of dalbavancin and for whom TDM sampling was logistically feasible according to the organizational workflow.

Written informed consent was obtained from the parents or legal guardians of all patients. This study was approved and registered by the local ethical committee (study number 678.430, approval protocol number 41193).

### 4.2. Treatment Protocol and Therapeutic Drug Monitoring

Patients received dalbavancin according to clinical needs, with dosing schedules individually tailored by the attending physician. In particular, dalbavancin was never prescribed as a routine first-line treatment. Its use was strictly reserved for selected patients requiring consolidation therapy to avoid prolonged hospitalization. In the majority of cases, it was employed for complex, deep-seated infections, often involving retained fixation devices or with inadequate source control, where a long-term intravenous regimen was clinically mandatory. In a minority of cases, it was indicated due to the unfeasibility of oral treatment because of poor adherence, intolerance, low oral bioavailability, or lack of effective oral alternatives according to antimicrobial susceptibility testing.

Patients with osteoarticular infections typically received a two-dose loading regimen (day 1 and 8) stratified by age (22.5 mg/kg for <6 years; 18 mg/kg for ≥6 years). Maintenance doses were subsequently administered at the same weight-based dosages at extended intervals (typically ranging from 4 to 6 weeks), guided by clinical stability and, when available, TDM. TDM samples were ideally collected at three key time points of each cycle: after drug infusion (C_max_), at an intermediate point (ideally 3 weeks post-dose), and immediately before the next administration (pre-dose). Throughout this study, both intermediate checks and strict pre-dose levels were collectively analyzed as trough concentrations (C_trough_) to characterize the entire elimination phase. However, the actual timing and frequency of sampling varied depending on the organizational workflow and outpatient visit scheduling.

Plasma concentrations were quantified using a validated liquid chromatography–tandem mass spectrometry (LC-MS/MS) kit provided by CoQua Lab (Turin, Italy), based on the method described by Mula et al. [38]. The assay was validated in accordance with international guidelines, ensuring linearity and precision over the clinical concentration range observed in this study.

### 4.3. Data Collection and Outcomes

All demographic, clinical, and microbiological data were retrieved from medical records. This study defined two co-primary outcomes tailored to the specific analysis sets as follows:

- Clinical primary outcome (total cohort): Clinical efficacy and safety. Clinical efficacy was defined as the complete resolution of clinical, hematological, and instrumental signs of infection with no recurrence during a follow-up period of at least 6 months;
- Pharmacokinetic primary outcome (TDM subgroup): An assessment of the pharmacokinetic feasibility of extending dosing intervals by analyzing the decay of plasma concentrations and identifying the duration of effective therapeutic coverage in a real-world pediatric setting.

As a secondary outcome, we performed a pharmacoeconomic analysis on the entire study population to evaluate healthcare resource utilization.

### 4.4. Statistical and Pharmacokinetic Analysis

Descriptive statistics summarized the cohort; clinical efficacy outcomes were reported as frequencies and percentages. In the TDM subgroup, Pearson’s correlation analyzed the logarithmic decay. Long-term pharmacokinetic stability and potential drug accumulation were evaluated using linear regression of trough concentrations against cumulative therapy duration, defined as the total number of days elapsed from the date of the first dalbavancin infusion to the date of the specific sample collection, encompassing multiple dosing cycles. A secondary analysis excluding Dose 1 was performed to mitigate sampling timing bias and to assess drug stationarity over the course of treatment. Samples were grouped by weeks elapsed since the previous administration (post-dose week); paired *t*-tests compared post-dose week 2 (reference) with subsequent time points to identify potential loss-of-coverage breakpoints. Logistic regression predicted sub-therapeutic exposure (trough concentrations <4 mg/L and <8 mg/L) adjusting for age, weight-adjusted dose, eGFR, duration of therapy, and time since last infusion; estimated marginal means (EMMs) projected the failure probabilities at mean intervals ±1 SD.

The pharmacoeconomic analysis evaluated hospitalization and CVC days saved by comparing observed length of stay (LOS) against the estimated LOS according to the standard of care (SoC) guidelines for the specific indication. In particular, the expected LOS was determined through an individualized assessment based on international guidelines. Specifically, the theoretical duration of standard intravenous therapy was tailored to each patient’s disease severity reflecting the actual hospitalization time that would have been clinically mandated for each specific patient.

Gross savings were estimated based on inpatient bed costs (EUR 688.50/day), whereas net savings factored in outpatient expenses (drug cost: EUR 388.17/vial; Day Hospital fee: EUR 340/visit). One-sample *t*-tests were used to test savings against zero.

All statistical analyses were performed using Excel (v16.0, Microsoft Corp., Redmond, WA, USA) and The jamovi project (2024). jamovi. (Version 2.6, Sydney, Australia) [Computer Software]. Retrieved from https://www.jamovi.org (accessed on 10 December 2025).

Statistical significance was defined as *p* < 0.05.

## 5. Conclusions

In conclusion, dalbavancin represents a valuable therapeutic strategy for pediatric staphylococcal infections, combining high efficacy with a favorable safety profile. Our PK analysis suggests that, while drug accumulation is not a concern, the maintenance of effective levels beyond the third week post-infusion may not be guaranteed. Therefore, TDM should be considered a fundamental tool to personalize dosing intervals in long-term multi-dose regimens, ensuring optimal drug exposure. Although our pharmacokinetic observations warrant validation in larger prospective studies, they provide a sufficient signal to propose a pragmatic management approach for maintenance regimens. If available, TDM should be performed around week 3 post-infusion to guide the timing of the subsequent dose. In the absence of TDM, we recommend that dosing intervals should not exceed 4 weeks to prevent the risk of sub-therapeutic exposure. The adoption of this precautionary approach, combined with the demonstrated economic benefits, supports a more extensive and rational use of dalbavancin in the pediatric setting.

## Figures and Tables

**Table 1:** Patient characteristics, dalbavancin dosing regimens, and TDM sampling distribution of the TDM subgroup.

	Patient			Dalbavancin Therapy		TDM Samples		
ID	Age (Years)	Weight (kg)	eGFR (mL/min)	No. of Doses (*n*)	Dalbavancin Administration (Weeks of Therapy)	Total Samples (*n*)	Peak Samples (*n*)	Trough Samples (*n*)
1	15	36	89.29	6	0, 2, 7, 14, 19, 25	12	3	9
2	9	27	144.50	5	0, 2, 6, 12, 16	10	4	6
3	9	29	132.82	3	0, 1, 7	5	2	3
5	14	46	142.56	5	0, 1, 7, 12, 17	10	4	6
6	16	47	143.18	4	0, 1, 9, 16	3	1	2
8	15	45	122.30	4	0, 1, 6, 11	8	3	5
9	12	67	157.29	3	0, 1, 6	6	3	3
11	15	54	111.73	8	0, 1, 4, 9, 14, 19, 24, 29	18	7	11
12	5	27	147.01	3	0, 1, 4	7	3	4

TDM: therapeutic drug monitoring; eGFR: estimated glomerular filtration rate. Peak samples: samples collected immediately at the end of infusion. Trough samples: samples collected during the elimination, including intermediate checks and pre-dose trough levels. Weeks of therapy: time points of drug administration expressed in weeks from the first dose (week 0).

**Table 2:** Descriptive statistics of dalbavancin plasma concentrations stratified by time elapsed since the last dose.

						Shapiro–Wilk	
	Mean (mg/L)	SD (mg/L)	CV (%)	Minimum (mg/L)	Maximum (mg/L)	*W*	*p*
Post-infusion	261.62	84.49	32.29	40.64	500.56	0.936	0.080
Post-dose 1 week	36.04	14.12	39.18	13.23	51.56	0.942	0.677
Post-dose 2 week	14.76	3.74	25.34	9.91	21.11	0.957	0.784
Post-dose 3 week	14.28	1.40	9.80	12.75	16.40	0.942	0.679
Post-dose 4 week	6.06	1.71	28.22	3.90	8.84	0.965	0.858
Post-dose 5 week	4.84	1.49	30.79	2.73	8.18	0.933	0.437
Post-dose ≥ 6 week	4.59	2.34	50.98	0.00	8.28	0.960	0.790

Data are presented as mean, standard deviation (SD), and range (minimum–maximum) for each time interval. Inter-individual variability is expressed as the coefficient of variation (CV%). The normality of distribution within each time window was assessed using the Shapiro–Wilk test (W), with *p*-values > 0.05 indicating a normal distribution.

**Table 3:** Statistical comparison of dalbavancin concentrations: Post-dose 2 weeks vs. subsequent time points.

		Statistic	*df*	*p*
Post-dose 2 week	Post-dose 3 week	0.224	2.00	0.843
	Post-dose 4 week	7.521	3.00	**0.005**
	Post-dose 5 week	8.172	4.00	**0.001**
	Post-dose ≥ 6 week	5.650	6.00	**0.001**

Paired samples *t*-tests were performed to evaluate differences in mean plasma concentrations between the reference stability period (post-dose week 2) and subsequent intervals within the dosing cycle. The table reports the t-statistic, degrees of freedom (*df*), and *p*-values (*p*) for each pairwise comparison. Statistically significant *p*-values (*p* < 0.05) are indicated in bold.

**Table 4:** Binomial logistic regression analysis of the factors associated with sub-therapeutic plasma concentrations (<4 mg/L).

						95% Confidence Interval	
	Estimate	SE	*Z*	*p*	Odds Ratio	Lower	Upper
Intercept	4.4306	10.0843	0.439	0.660	83.985	2.19 × 10^−7^	3.22 × 10^10^
Age (years)	0.0348	0.3362	0.103	0.918	1.035	0.536	2.00
Dose (mg/kg)	−0.3058	0.2058	−1.486	0.137	0.737	0.492	1.10
Days of therapy (days)	−0.0345	0.0208	−1.653	0.098	0.966	0.927	1.01
C_trough_ timing (weeks)	1.0862	0.4445	2.444	**0.015**	2.963	1.240	7.08
eGFR (mL/min)	−0.0334	0.0415	−0.805	0.421	0.967	0.892	1.05

Statistically significant *p*-values (*p* < 0.05) are indicated in bold.

**Table 5:** Binomial logistic regression analysis of the factors associated with sub-therapeutic plasma concentrations (<8 mg/L).

						95% Confidence Interval	
	Estimate	SE	*Z*	*p*	Odds Ratio	Lower	Upper
Intercept	−1.53561	7.99979	−0.1920	0.848	0.215	3.34 × 10^−8^	1.39 × 10^6^
Age (years)	−0.07252	0.19706	−0.3680	0.713	0.930	0.632	1.37
Dose (mg/kg)	−0.10042	0.20949	−0.4794	0.632	0.904	0.600	1.36
Days of therapy (days)	0.00309	0.00985	0.3133	0.754	1.003	0.984	1.02
C_trough_ timing (weeks)	1.11440	0.32754	3.4023	**<0.001**	3.048	1.604	5.79
eGFR (mL/min)	−8.04 × 10^−4^	0.03279	−0.0245	0.980	0.999	0.937	1.07

Statistically significant *p*-values (*p* < 0.05) are indicated in bold.

**Table 6:** Descriptive and inferential statistics regarding clinical resource and economic savings (*n* = 16).

			Percentiles		One-Sample *t*-Test	
	Median	Min–Max	25th	75th	*t*-Value	*p*-Value
Hospitalization days saved	8.50	0–19	5.00	8.50	*t* = 5.74	**<0.001**
CVC days saved	6	0–10	1.00	6	*t* = 3.59	**0.009**
Hospitalization cost saved (€)	5852.25	0–13,082	3442.50	5852.25	*t* = 5.74	**<0.001**
Final net saving (€)	3215.84	−6358–7840	−738.79	3215.84	*t* = 3.45	**0.004**

One-sample *t*-test was performed to test if savings were significantly greater than 0. Statistically significant *p*-values (*p* < 0.05) are indicated in bold.

## Data Availability

The data presented in this study are available upon request from the corresponding author. The data are not publicly available due to privacy restrictions.

## Supplementary Materials

The following supporting information can be downloaded at: https://www.mdpi.com/article/10.3390/antibiotics15020162/s1. Table S1: Demographic, clinical, microbiological, and therapeutic characteristics of 16 patients treated with dalbavancin.

## Abbreviations

The following abbreviations are used in this manuscript:

ABSSSI	Acute Bacterial Skin and Skin Structure Infection
AE	Adverse Event
ALL	Acute Lymphoblastic Leukemia
CHD	Congenital Heart Disease
CI	Confidence Interval
Cmax	Peak Plasma Concentration (Post-Infusion)
CRBSI	Catheter-Related Bloodstream Infection
Ctrough	Trough Plasma Concentration
CV	Coefficient of Variation
CVC	Central Venous Catheter
df	Degrees of Freedom
eGFR	Estimated Glomerular Filtration Rate
EMA	European Medicines Agency
EMMs	Estimated Marginal Means
FDA	Food and Drug Administration
IQR	Interquartile Range
IV	Intravenous
LC-MS/MS	Liquid Chromatography–Tandem Mass Spectrometry
LOS	Length of Stay
MIC	Minimum Inhibitory Concentration
MRSA	Methicillin-Resistant *Staphylococcus aureus*
MSSA	Methicillin-Susceptible *Staphylococcus aureus*
NA	Not Available
OAI	Osteoarticular Infection
OR	Odds Ratio
PD	Post Discharge
PJI	Prosthetic Joint Infection
PK	Pharmacokinetics
PK/PD	Pharmacokinetic/Pharmacodynamic
Pt	Patient
PVL	Panton–Valentine Leucocidin
SD	Standard Deviation
SE	Standard Error
SoC	Standard of Care
TDM	Therapeutic Drug Monitoring
