# Supplementary material for: TDM-Guided Dalbavancin Treatment for Complex Staphylococcus aureus Osteoarticular Infections in Children
Source: Antibiotics (Basel). 2026 Feb 3;15(2):162. doi: 10.3390/antibiotics15020162 (PMC12937467; doi:10.3390/antibiotics15020162)
Supplement: Supplementary file 1 [file antibiotics-15-00162-s001.zip › antibiotics-4101266-supplementary.pdf]

Table S1: Demographic, clinical, microbiological, and therapeutic characteristics of 16 patients treated with dalbavancin.

Patient			Infection					Previous anti-Gram-positive therapy		Dalbavancin				
**ID**	**Age/ sex**	**Comorbidities**	**Diagnosis**	**Prosthetic device***	**Pathogen**	**PVL**	**MIC V/T**	**Days**	**Antibiotics**	**Doses (*n*)**	**Dosage (mg/kg)**	**Dalbavancin from discharge** (days)**	**Side effects**	**Clinical outcome**
							**BONE AND JOINT INFECTION**							
1	15/M	Down Syndr., CHD, Epilepsy	Post-fracture osteomyelitis (Femur)	Yes	MRSA, *K. pneumoniae*, *E. faecalis*	Neg	1/<0.5	44	Beta-lactams, Glycopeptides, Daptomycin	6	17.5	1	None	Success
2	9/M	None	Post-fracture osteomyelitis (Elbow)	No	MRSA	Neg	1/<0.5	31	Glycopeptides, Daptomycin, Fosfomycin, Rifampicin	5	17.4	PD	None	Success
3	9/F	None	Chronic osteomyelitis (Tibia)	Yes	MSSA	Pos	1/<0.5	14	Beta-lactams, Clindamycin, Daptomycin	2	18.00	0	None	Success
5	14/M	Genetic syndrome, Epilepsy	PJI (Vertebrae)	Yes	MSSA	Neg	2/<2	23	Beta-lactams, Daptomycin, Fosfomycin	5	18.48	0	None	Failure
6	16/F	Osteosarcoma	PJI (Knee)	Yes	MRSA	Neg	1/<0.5	31	Glycopeptides, Daptomycin, Fosfomycin	4	18.09	0	None	Success
7	1/F	None	Sepsis and Osteomyelitis (Humerus)	No	MSSA	Pos	<0.5/<0.5	36	Beta-lactams, Glycopeptides, Daptomycin, Linezolid	2	22.33	0	None	Success
8	15/M	Genetic syndrome, Epilepsy	PJI (Vertebrae)	Yes	MSSA	Neg	2/<2	13	Daptomycin, Fosfomycin	4	18.48	0	None	Success

9	12/F	None	Sepsis with OA dissemination	No	MSSA	Pos	1/<0.5	47	Beta-lactams, Glycopeptides, Clindamycin, Daptomycin, Linezolid, Fosfomycin	3	15.20	0	None	Success
10	16/M	None	PJI Prophylaxis (Hip)	Yes	MSSA	Pos	2/<0.5	1	Beta-lactams	2	14.49	0	None	Success
11	15/F	None	Post-fracture osteomyelitis (Femur)	Yes	*S. condimenti*	NA	1/NA	17	Beta-lactams, Clindamycin, Daptomycin	8	18.52	0	None	Success
12	5/F	None	Osteomyelitis (Femur)	No	MRSA	Neg	<0.5/<0.5	18	Glycopeptides, Daptomycin, Fosfomycin, Rifampicin	3	22.22	1	None	Success
13	3/M	None	Post–partial amputation osteomyelitis (Leg)	Yes	Unknown	-	-	32	Glycopeptides, Daptomycin	3	23.53	0	None	Success
							**ABSSSI**							
4	17/M	Autoimmune polyradiculoneuritis	Soft tissue infection (paranasal)	No	MSSA	Neg	<0.5/<0.5	1	None	1	18.18	0	None	Success
14	12/F	None	Complicated pyoderma	No	MRSA	Pos	1/<0.5	7	Beta-lactams, Glycopeptides, Clindamycin	2	19.12	1	None	Success
16	9/M	None	Surgical site infection	Yes	Unknown	-	-	13	Glycopeptides	1	17.50	0	None	Success
							**BLOODSTREAM INFECTION**							
15	4/F	ALL	Complicated CRBSI	No	MSSA	Neg	<0.5/<0.5	31	Beta-lactams, Glycopeptides, Daptomycin	1	23.33	2	None	Success

PVL: Panton-Valentine Leucocidin; MIC: Minimum Inhibitory Concentration; V: Vancomycin; T: Teicoplanin M: Male; F: Female, CHD: Congenital Heart Disease; MRSA: Methicillin-Resistant Staphylococcus aureus; MSSA: Methicillin-Susceptible Staphylococcus aureus; PJI: Prosthetic Joint Infection; OA: Osteoarticular (dissemination); ALL: Acute Lymphoblastic Leukaemia; ABSSSIs: Acute Bacterial Skin and Skin Structure Infections; CRBSI: Catheter-Related Bloodstream Infection, PD: post discharge, NA: not available.

Notes:

**Prosthetic device: Refers to the presence of orthopaedic devices (e.g., prosthetic joints, internal fixation plates). **Time to Disch. (Time to Discharge): Calculated as the number of days elapsed between the first dose of dalbavancin and hospital discharge. A value of 0 indicates that dalbavancin was administered on the day of discharge.*
